# Blending and Conversion of Types in Fevers

**Published:** 1853-02

**Authors:** 


### SELECTIONS.

[We are indebted to the *Am. Journal of the Med. Sciences*, for the following notice of Dr. Dickson's Report to the American Medical Association, "On the Blending and Conversion of Types in Fever."

Our Philadelphia friends are favored in living nearer the Orient, but we live in hope. We confess that we see no reason why it should take some months for the *Transactions* to reach us, while the *Journals* come in a few days.—Eds.]

Dr. Dickson has treated the subject referred to him in a very masterly manner; his general conclusions are cautiously drawn, and bear the marks of truth; while he admits that the types of fever of a very dissimilar origin and character may become blended together in the same case, and that one type may be substituted for another; he denies that the *conversion*, strictly speaking, of one type of fever into another can, under any circumstances, take place.

Dr. Dickson refers to the very loose and indefinite manner in which the term type, when applied to fevers, has been employed even by the most distinguished medical authors. It has been made use of to express promiscuously all the varied relations of this class of diseases, "some of which are those of strong resemblance and close affinity, others again of marked dissimilarity, and others still of almost absolute contrast." Although not prepared to offer exact limitations to the use of the word type, it must, he considers, be understood always to convey some marked distinction, lying deeper than the general relations that connect the subjects treated of. While, he remarks, we may still dispute whether typhus and typhoid fevers differ essentially, no one will confound scarlatina or a tertian intermittent with either.

"We are persuaded," he says, "that the truth will, on examination, be found in the following propositions: 1. That each type, or marked variety of fever, is the result of a definite cause, relevant in its properties, characters, and mode of action, to the effects produced, however obscure this relevancy may be, and ill understood, in the present state of our knowledge. 2. That these causes, varying greatly in nature, must be sometimes similar, sometimes dissimilar, and sometimes contrasted or opposite in the character or mode of their efficiency. 3. That causes of different kinds may sometimes coexist. 4. That when they resemble each other, their effects or influences are really *blended*, and mingled, and interchanged, as one or the other may predominate. 5. That when dissimilar causes coexist, they may sometimes act together, but not often; may sometimes blend their influences, but not readily or freely; they may possibly supersede each other by *substitution*, but in no imaginable instance can the effect of one cause be the effect of another and dissimilar cause. This sort of transformation, the only true conversion in the logical sense, is a rational impossibility, whether we regard diseases materially and ontologically as entities, or pathologically and dynamically as mere affections of the organism arising out of precedent impressions from causative agents. We do not deny the difficulty of distinguishing practically, such substitution for conversion so called, we desire only to express our meaning precisely, in order that our views may be clearly understood.

"The records before us present numerous examples of the *blending* of types of fever—the intermingling of characteristic features. This is a phenomenon by no means rare; it is, for the most part, easily explained, and the coexistence of more than one cause may usually be indicated to account for it.

"There are also abundant instances, related upon sufficient authority, in which one form or type takes the place of another which has preceded it. This, which we shall describe and comment upon, is the *conversion* of common phraseology. We have said that we believe it to be correctly substitution, and not transformation; but we have no objection to the employment of the above term, so convenient, so much more familiar, and, indeed, so indicative of the change that has occurred."

Dr. Dickson supposes, therefore, some special relation to exist among fevers apparently derived from the same source that implies their convertibility, and believes that it is just as reasonably to be inferred that fevers thus connate are *exclusively* interchangeable; those which arise from distinct causes, not being thus related, are not convertible.

The periodical fevers, which are referable to a common origin, and connected by a history of common properties, are referred to as notoriously mutable or convertible; the remittent may subside into an intermittent, the intermittent being aggravated into a remittent, a quotidian falling into a tertiary or a quartan, and these becoming duplicated, complicated, or exasperated into quotidian frequency.

Continued fevers, while they are strongly contradistinguished from the periodical by many remarkable points in their history and character, are, also, closely allied to each other, so that the true diagnosis of the several varieties distinguished by name is still warmly disputed. These, likewise, mingle and run into one another by blending and interfusion, as might fairly be inferred from the prolonged disputes as to their identity, and differences, and mutual relations, whether of resemblance or contrast.

"The exanthemata," Dr. Dickson remarks, "form a class of fevers which, at first sight, would appear to be palpably and obviously separable from all other types. But a more careful examination will show that even here it is not easy to establish entirely, and preserve uninfringed, clear boundary lines between neighboring and connate maladies. The characteristic features, indeed, of the eruptive fevers are so far unsettled, that we find pathologists of high reputation including under this head all the continued and some of the uncertain types. Both typhoid and true typhus have been arranged here; yellow fever, which has been assigned a position on almost every column of the nosological catalogue, is pronounced by Hildenbrand to be an exanthem; cholera asphyxia is thus regarded by Parke, Simon, and Horner; and dengue—by some merged into the ranks of the malarious remittents, by others classed with yellow fever, and by Cooke and Copeland recognised as a variety of scarlatina—is, to say the least, more closely affiliated here than anywhere else."

Dr. Dickson assumes the establishment of the following distinct types of fever :—

"1. The periodical; including (after Bartlett) the intermittent, remittent, and congestive.

"2. The continued; comprising typhoid, true typhus, simple fever, ephemera, febriculæ, British epidemic fever, relapsing fever.

"3. The exanthematous; variola, scarlatina, measles, dengue.

"4. Yellow fever; the hæmagastric pestilence of Copland, causus of Mosely, typhus icterodes of Cullen, malignant remittent of Rush.

"5. Catarrhal fever, known when epidemic as influenza."

The question as to the blending of these several types is very fully and satisfactorily discussed. The subject of the apparent conversion of the types of fever is next considered.

"The examples above given of the mingling and interfusion of symptoms of two or more forms of fever," says Dr. Dickson, "differ among themselves, in the greater predominance of one morbid influence or another in the several cases. Of numerous attacks which commence in the same way, the course, history, and ultimate termination may be strikingly diverse. Invading with the ordinary symptoms of climatorial, autumnal bilious fever, one shall retain its periodical malarious character, ending as it begun, with the features of a simple remittent. Another, losing these features in a protracted course, shall grow more and more continuous, and, ultimately, put on all the appearances of maculated typhus, with dry, dark tongue; mouth, teeth, and gums blackened with sordes; or present the meteorism and abdominal disorder of typhoid, with intestinal ulceration shown *post mortem*; and a third shall sink promptly into profound collapse, dying with orange-yellow discoloration of skin and eyes, and black vomit. If we suppose all these to have been struck down by malaria, in a locality infected by the coexistence of the three morbid poisons, it is easy to imagine some of them to come under the influence of ochlosis, the alleged source of typhus and typhoid, and others, strangers and predisposed, to fall victims to the obscure cause of the hæmagastric pestilence. We may correctly assume that, in all these, a temporary *blending* of types took place at a certain stage of their progress, under the conjoint influence of the several concurrent causes. As they advanced, however, the more intense or forcible influence would predominate; generally speaking, the malarious characteristics, periodicity, especially, would disappear, being substituted or supplanted by those of the more malignant poisons; a virtual and complete *conversion of type.* In this sense, then, we believe that conversion is not only possible, but frequent, and shall proceed to adduce in proof a few additional evidences."

One more extract from this interesting report must suffice; all we desire being to present to our readers a fair exposition of the general positions assumed by the author, in reference to the blending and conversion of types in fever.

"If," Dr. D. remarks, "contagious diseases can be originated or generated under any contingency whatever, and no one can doubt the possibility of this occurrence, and if the matter of contagion is, as indeed it must be, a vital individuality, a self-multiplying germ, capable of indefinite reproduction, then the creation or development of this new cause must give rise to new results. A new form of disease now presents itself, which either *blends* with that which pre-existed, or supplants and *substitutes* it. If the causes of the two are connate and correlated, and in any degree similar or analogous in influence, *blending of type* takes place; the more especially if they are nearly equal or quite equal in force, and circumstances do not strongly favor either at the expense of the other. But if they be markedly dissimilar or contrasted, or in any manner incompatible, or if circumstances favor the one and repress the other, then there will and must happen the subversion of the first, the weaker or least tenacious; and *conversion of type* will take place, in the only sense possible and intelligible."

